# FEASIBILITY AND ACCEPTABILITY OF IMPLEMENTING AN AI-ENABLED SERVICE ROBOT IN LONG-TERM CARE

**DOI:** 10.1093/geroni/igae098.0115

**Published:** 2024-12-31

**Authors:** Lillian Hung, Haopu (Lily) Ren, Karen Lok Yi Wong, Katharine Davies, Arisa Kinugawa

**Affiliations:** University of British Columbia, Vancouver, British Columbia, Canada; University of British Columbia, Vancouver, British Columbia, Canada; University of British Columbia, Vancouver, British Columbia, Canada; University of British Columbia, Vancouver, British Columbia, Canada; University of British Columbia, Vancouver, British Columbia, Canada

## Abstract

In long-term care (LTC), staff members are responsible for the complex needs of people with disabilities. This task can be enhanced by integrating service robots equipped with Artificial Intelligence (AI). These robots offer personalized service for residents and empower the staff with efficient support. Despite growing interest, the implementation of service robots in LTC remains under-investigated. This study examines the feasibility and acceptability of implementing a service robot, named Aether, in a Canadian LTC home, guided by the underpinnings of Collaborative Action Research. We included interdisciplinary staff in pre- and post-intervention focus groups and conducted conversational interviews with residents, staff members, and managers. Consolidated Framework for Implementation Research (CFIR) informed our implementation, data collection and analysis. We identified key facilitators (staff engagement and training) and barriers (environmental dynamics and resource limitations). Our results underscore the imperative of structural support at micro-, meso- and macro-levels for staff in LTC to implement technology effectively. This study contributes valuable insights into the future development and deployment of AI-enabled service robots in LTC.

